# Recognizing Descending Necrotizing Mediastinitis in Adults: A Narrative Review of Diagnostic Dilemmas and Barriers

**DOI:** 10.7759/cureus.112582

**Published:** 2026-07-13

**Authors:** Christina Avgoustou, Constantinos Avgoustou

**Affiliations:** 1 Fourth Department of Internal Medicine, National and Kapodistrian University of Athens, Athens, GRC; 2 Department of Surgery, General Hospital of Nea Ionia "Konstantopouleio", Athens, GRC

**Keywords:** deep neck infection, descending necrotizing mediastinitis, diagnostic challenges, multidisciplinary approach, prompt evaluation

## Abstract

Unrecognized, untreated, or treatment-resistant deep neck infections (DNIs), commonly originating from oropharyngeal or odontogenic sources, can rapidly lead to life-threatening complications such as airway obstruction, systemic sepsis, and descending necrotizing mediastinitis (DNM). Severe infections may spread downward from the head and neck through and along fascial planes into the mediastinum. This process continues to be associated with high mortality rates and severe associated morbidity. Early diagnosis remains particularly difficult and challenging, and delayed recognition is linked to poor clinical outcomes. However, modern, tailored multidisciplinary management approaches, including prompt diagnosis and timely, coordinated medico-surgical treatments, have improved survival rates. Optimizing clinical outcomes is fundamentally time-dependent, necessitating earlier recognition of the disease and faster initiation of effective therapeutic interventions. This review highlights key considerations for ensuring an efficient and timely diagnostic evaluation throughout the progression of this potentially fatal infection.

## Introduction and background

Deep neck infection (DNI) refers to the infectious process involving the fascial layers (namely, the sheets of connective tissue supporting and separating body structures) and spaces beneath the superficial cervical fascia and the filling tissues. If left untreated or not adequately treated, these infections may progress to necrotizing fasciitis (NF) that involves the head and/or neck, a severe condition that can lead to airway obstruction [1,2]. First descriptions of NF date back to Hippocrates in the 5th century B.C. An especially feared and highly morbid and lethal complication of head and neck NF (H&N NF) is descending necrotizing mediastinitis (DNM), which is the rapid downward spread of infection through and along fascial planes of the neck into the mediastinum and thoracic cavities. DNM is a relatively uncommon infection and typically polymicrobial, arising from various oropharyngeal and cervical infectious sources [3,4]. This infection is characterized by high virulence and a fulminant progressive course, often resulting in sepsis, multiple organ failure, and ultimately death [4-6].

The proximity and complexity of the anatomic structures of the neck, combined with the aggressive nature of the synergistic pathogens involved, pose a significant challenge in the management of DNIs/DNM [3,7]. More precisely, the often mild and insidious onset of the infection frequently delays diagnosis, while its rapid progression can lead to severe complications affecting vital structures [3,4]. To compound matters, surgical drainage procedures may occasionally be insufficient, further contributing to poor outcomes [8,9]. Specifically for DNM, sub-optimal surgical management can occur regardless of the choice between video-assisted thoracoscopic surgery and thoracotomy. As a consequence, despite advances in diagnostic methods and the development of tailored, authoritative treatment strategies, DNM remains a life-threatening condition with mortality rates that can still reach up to 40%, largely due to sepsis and multiple organ dysfunction or failure [2,3,8,10].

Existing literature supports the critical importance of rapid and accurate diagnostic and therapeutic strategies for DNI/DNM. Accordingly, improved outcomes have been reported in DNM patients treated promptly and aggressively in specialized centers where coordinated multispecialty care is available [3,11]. Nevertheless, owing to the low incidence of this pathology and the challenges inherent in differentiating it from its mimics, definitive diagnosis and targeted management are frequently delayed or suboptimally executed. Furthermore, a definitive consensus regarding an optimal management protocol has yet to be reached: current therapeutic frameworks are largely limited to preliminary recommendations rather than well-validated clinical guidelines, and they are substantially extrapolated from management protocols established for necrotizing soft-tissue infections at alternative anatomical sites [12]. To increase clinical awareness and vigilance regarding these frequently underestimated but life-threatening infections, this review focuses on critical aspects of modern diagnostic evaluation highlighted by recent landmark studies. Prior to this diagnostic overview, a concise analysis of the core anatomical and pathophysiological landmarks relevant to the disease is provided to facilitate a deeper understanding of its clinical progression.

## Review

Methods

Our search of the literature was conducted during March 2026 and included an electronic search across the PubMed/Medical Literature Analysis and Retrieval System Online (MEDLINE) database. The literature search encompassed articles published from January 2011 through February 2026. Since our primary aim was to assess the recent trends and pitfalls regarding the diagnosis of DNM, occurring in the context of a head and neck infection, our search strategy included articles referring either to DNM or to H&N NF. We evaluated studies regarding the definition of the diseases, and we included only those with widely accepted criteria of diagnosis for H&N NF and DNM: specifically, both suggestive imaging findings and tissue pathology or definite operative findings consistent with NF and DNM were required, whether described circumlocutorily or via well-established diagnostic criteria. For H&N NF, abstracts were reviewed to determine whether the report included mention of both NF and DNM.

The specific search terms used were: (“Mediastinitis” OR “descending necrotizing mediastinitis” OR “DNM”) AND “Mortality"; “Descending necrotizing mediastinitis” AND (“Diagnosis” OR “Algorithm”); “Descending necrotizing mediastinitis” AND (“Imaging” OR “CT” OR “MRI”); “Descending necrotizing mediastinitis” AND (“Approach” OR “Assessment” OR “Management”); “Head and neck necrotizing fasciitis” AND “Mortality”; “Head and neck necrotizing fasciitis” AND "Diagnosis".

Other closely related terms used to broaden the identified literature included "descending cervical mediastinitis (DCM)," "necrotizing fasciitis of the neck," "cervical necrotizing fasciitis (CNF)," “cervicofacial necrotizing fasciitis," and “craniocervical necrotizing fasciitis."

Eligibility Criteria

Studies were selected based on specific, predefined inclusion and exclusion criteria. Inclusion criteria consisted of original peer-reviewed research articles (prospective or retrospective cohort studies and case series with at least three patients); studies evaluating adult populations diagnosed with H&N NF or DNM; studies published in the English language; and manuscripts on human subjects.

Exclusion criteria consisted of case reports involving fewer than three patients, conference proceedings, editorials, systematic reviews, meta-analyses, and expert opinions; studies published in languages other than English; and animal models or in vitro experimental studies.

Study Selection and Data Extraction

Two independent reviewers screened the titles and abstracts of all retrieved citations against the eligibility criteria. Discrepancies regarding study inclusion were resolved through a consensus process. Data regarding study design, sample size, diagnostic protocols, and clinical outcomes were subsequently extracted and synthesized qualitatively in this narrative review.

The collected research on diagnostic approaches and mortality data of DNM revealed an absolute predominance of case reports or case series retrospectively analyzed. The reference lists of all retrieved articles were manually screened to identify any further relevant studies that might have been omitted during the initial electronic search. Through this manual screening, selected landmark or highly illustrative case reports, and reviews (systematic or narrative) offering significant clinical insights into atypical presentations, diagnostic pitfalls, or novel therapeutic approaches were subsequently included.

Neck anatomy and disease pathophysiology

Approximately 3-10% of all NF cases involve the head and neck region. These cases present distinctive diagnostic and therapeutic challenges because of the complex anatomical framework of this area and its close proximity to the upper airway tract [13]. Around 1% of severe odontogenic infections progress to NF, while 1.5-3.6% of DNIs advance to DNM [10,13-15].

**Figure 1:**
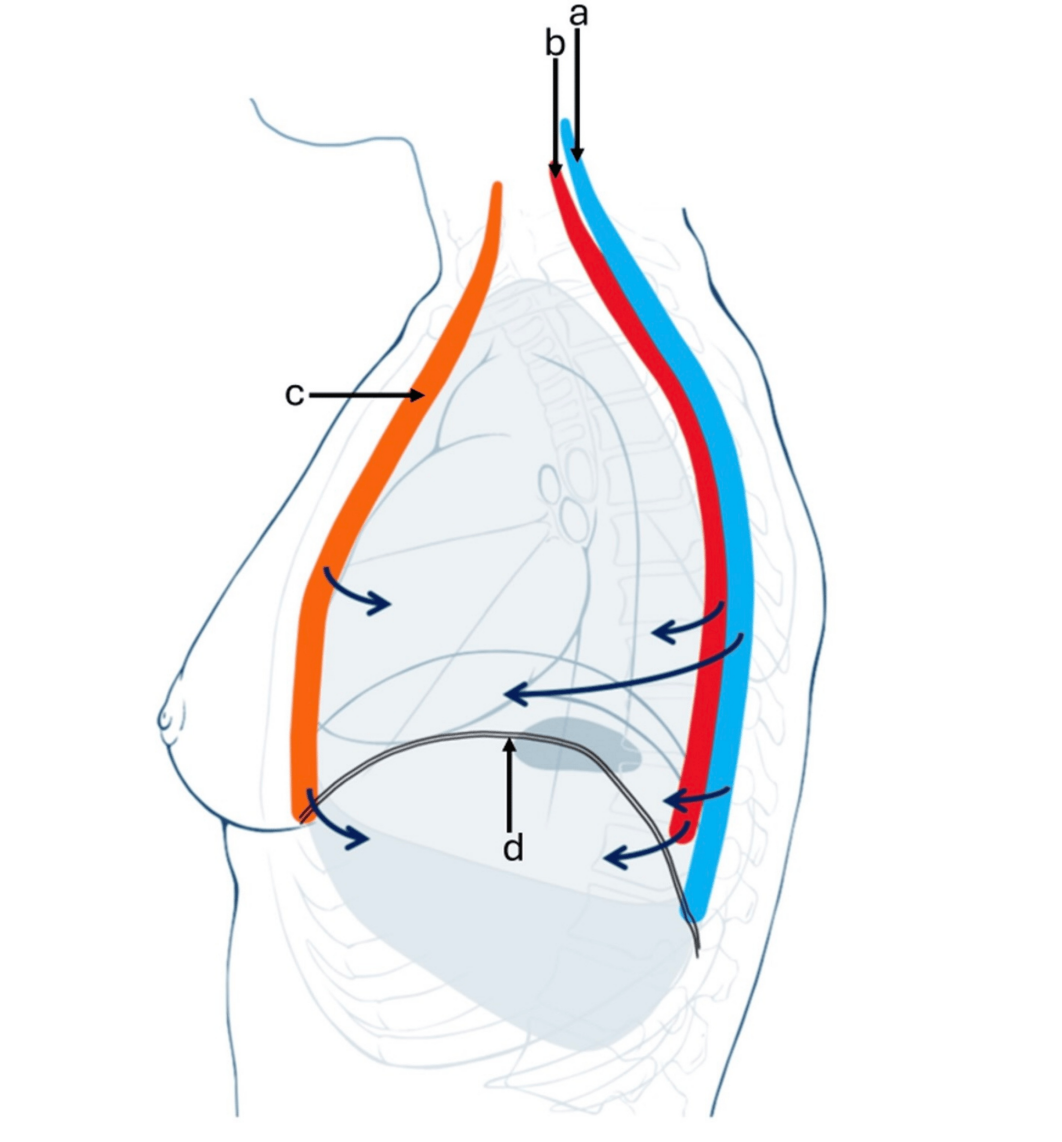
Deep spaces of the neck and their communications with the chest a: prevertebral; b: retrovisceral; c: pretrachial; and with d, the diaphragm is indicated. Adapted by the author from "Lynch - Drawing Lateral chest landmarks - no labels" by Patrick J. Lynch and C. Carl Jaffe, license: CC BY 4.0 [16].

A comprehensive understanding of the anatomy and function of the cervicomediastinal fascial spaces and planes is essential for both the diagnosis and surgical management of DNIs/DNM [3]. Briefly, the deep cervical fascia is divided into three layers: the superficial, the visceral, and the prevertebral; these layers separate the neck into three principal compartments: the pretracheal, retrovisceral, and perivascular (Figure 1) [16]. Each of these compartments can serve as a portal of entry and pathway for the spread of infection into the mediastinum and, in rare cases, even into the abdominal cavity [4,17]. More precisely, the pretracheal fascia has attachments to the parietal pleura and the pericardium, providing a potential route for downward extension of infection. This anatomical relationship accounts for the frequent occurrence of pulmonary empyema and purulent pericarditis associated with DNM [18]. The retrovisceral space is the most common pathway through which oropharyngeal infections spread. Infections may also track along vascular structures within the perivascular compartment, such as the carotid sheath, towards the mediastinum and pleural cavities [18]. The parapharyngeal space represents a pivotal route within the neck, communicating with all major fascial spaces.

Clinical progression is characterized by a sequential infectious process extending through contiguous tissue planes: untreated odontogenic, peritonsillar, and oropharyngeal infections may lead to abscess formation and, in rare cases, progress to the potentially fatal condition of cervical NF [1,19]. Odontogenic sources are identified as the most frequently reported cause for such complications in recent studies. During the early stages of NF, the overlying tissues often appear unaffected, which may significantly delay both diagnosis and surgical intervention [19]. As the disease progresses, the infection can extend beyond the fascial layers to involve subcutaneous and soft tissues and even muscle structures [13]. Finally, cervical NF will be characterized by fulminant and aggressive progression with widespread tissue necrosis [18]. The downward spread of infection to the mediastinum, leading to DNM, follows the specific anatomical routes described above and is mediated by several factors, such as gravity, respiration, the negative intrathoracic pressure in the mediastinum and pleural cavities during inspiration, and the absence of barriers within the fascial structures [10]. Notably, in rare cases, thyroid abscesses and post-thyroidectomy site infections may also extend into the mediastinum and cause DNM. However, the unique anatomical and physiological features of the gland, including its rich blood supply and lymphatic drainage, high iodine content, and the presence of a thick protective capsule, render the thyroid gland resistant to infections [20,21]. Comorbid conditions that impair immune function or reduce tissue oxygenation can predispose patients to the development and facilitate the progression of such infections [4,10]. Finally, fulminant extrathoracic sequelae can develop secondary to DNI/DNM, significantly compromising clinical outcomes and elevating mortality. These include Ludwig's angina, a complication of severe oropharyngeal infection characterized by edema beneath the tongue and in the neck that requires urgent management [15,22]; Lemierre’s syndrome, resulting from oropharyngeal infection and manifesting as internal jugular vein thrombophlebitis and septicemia [10,15]; and orbital involvement, which significantly increases morbidity and mortality [13].

The pathological features of cervical NF and DNM typically include tissue edema (e.g., cervical and esophageal edema, pneumonitis), fluid accumulation (e.g., pleural and pericardial effusions), abscess formation, liquefactive necrosis, and tissue air infiltration [14]. Regional tissue edema may become so severe that airway management becomes extremely difficult, particularly in the event of accidental dislodgement or extraction of the endotracheal tube [18].

From a microbiological perspective, organisms that normally constitute the commensal oropharyngeal and dental flora can act synergistically to become highly invasive and virulent, thereby precipitating the systemic inflammatory response [15]. In most cases, the infection is polymicrobial, with cultures commonly demonstrating a mixture of anaerobic and aerobic microorganisms [8].

Diagnosis

Clinical Presentation and Physical Examination Findings

In the early stages, a cervicopharyngeal infection that progresses to DNI is often clinically subtle and may remain relatively silent, probably veiled by analgesic use that masks symptoms, further delaying diagnosis [10]. Early manifestations are usually nonspecific and may include neck pain (often following a toothache after tooth extraction or an abscess), fever, otalgia, toothache, odynophagia, dysphagia of both solids and liquids, and speech difficulties such as dysphonia/hoarseness associated with “sore throat” [7,9,10,22]. Foreign body sensation and upper back and/or shoulder pain have also been described. If the condition remains untreated or is inadequately managed, it may rapidly progress to descending suppurative mediastinitis. This condition typically manifests 12 hours to two weeks after initial infection (average delay of seven days), with a more aggressive reported course (less than 24 hours) in the case of DNM [8,23,24]. At this stage, more severe manifestations may develop, reflecting the disease worsening, including dyspnea, high fever with chills, retrosternal chest pain, hemodynamic instability, headache, and worsening of general condition [9,13].

Physical examination findings may include cervical erythema, tenderness, and localized swelling (e.g., peritonsillar abscess, Ludwig’s angina), lymphadenopathy, neck stiffness (torticollis), trismus, stridor, bulging of the posterior pharyngeal wall, edema of the oropharyngeal structures, asymmetry of the neck, and swelling of the lower neck and upper chest. Additionally, advanced signs such as swelling of the epiglottis, narrowing of the glottic space, subcutaneous emphysema (crepitus or audible “crunching” sound on auscultation), and fistulization of the aerodigestive tract to the skin may be encountered [7,8,15]. Among all these, respiratory distress is the most critical finding, making timely airway safeguarding the most urgent and essential intervention [9]. Respiratory function can be compromised early in the disease course, even when the infection initially presents as confined localized DNI, due to excessive cervical swelling caused by purulent accumulations in the neck [3]. In cases of Ludwig’s angina, the associated life-threatening edema may necessitate immediate airway intervention, with up to 70% of patients requiring prompt surgical opening [10,22]. Occasionally, even at the stage of mediastinitis, the extent of infection is often disproportionate to the apparent clinical signs and symptoms [15]. Unlike NF in other anatomical regions, such as the trunk, extremities, external genitalia, and perineum, where severe pain disproportionate to local inflammatory findings is a prominent feature, in H&N NF, this symptom may be overshadowed by the more immediate and severe condition of respiratory compromise [1,25]. Septic shock accompanying the diagnosis of DNM is relatively common, with reported frequencies ranging from 13% to 55.5% [11,15,26,27]. Additional manifestations of systemic toxicity, including acute respiratory distress syndrome (ARDS), acute kidney injury, and multiple organ dysfunction or failure, predominantly occur in patients with delayed hospital presentation or protracted clinical management [17,22].

In the most severe disease, major complications may manifest alongside their associated clinical findings. Several vascular complications have been reported, such as internal jugular vein thrombophlebitis (Lemierre’s syndrome) with septicemia, erosion or rupture of the carotid or other vessels leading to massive hemorrhage, intrathoracic bleeding, cardiac tamponade, and disseminated intravascular coagulation [15,17,22]. More rarely, neurological complications such as cranial nerve paresis (e.g., involving orbital nerves, brachial plexus, and recurrent laryngeal nerve), brain abscess, meningitis, and vertebral osteomyelitis may also develop [2,4,25,28]. Pneumomediastinum (mediastinal emphysema) as well as subcutaneous emphysema are rare but potentially life-threatening complications. These conditions are sometimes associated with the use of high-speed air-driven handpieces during dental procedures, such as third molar extraction, which may introduce air directly into the fascial planes [28]. However, gas formation can also result from the activity of synergistic microorganisms responsible for rapidly spreading necrotizing infections [8,28]. Several regional sequelae, including cardiac arrhythmias, pericarditis, and salivary fistulas, can also emerge. Orbital involvement and skin necrosis, when present, are particularly serious and are associated with significantly increased morbidity and mortality [13].

Laboratory Assessment

Although laboratory findings are non-diagnostic, they critically support the diagnostic process and guide subsequent clinical management. Pertinent parameters include leukocytosis, elevated serum creatinine, hypoalbuminemia, dysregulated blood glucose levels, thrombocytopenia, and anemia. Hyponatremia and altered chloride levels, alongside metabolic acidosis, elevated lactate, and increased creatine kinase, may also be observed. Furthermore, inflammatory markers such as C-reactive protein and procalcitonin, as well as shifts in neutrophil and lymphocyte counts and their corresponding ratios, assist both in suspecting the diagnosis and tailoring antimicrobial therapy [19].

Several laboratory-based scoring systems are commonly used in clinical practice to help discriminate necrotizing infections from non-necrotizing infections (e.g., cellulitis) and to estimate disease severity and survival probability, although their diagnostic accuracy is not always unequivocal [10]. Among the most widely used scoring tools are APACHE II (Acute Physiology and Chronic Health Evaluation), SAPS-3 (Simplified Acute Physiology Score), and SOFA (Sequential Organ Failure Assessment), including its variants such as nfSOFA (SOFA score for NF) and qSOFA (quick SOFA). Another frequently applied tool is the LRINEC (Laboratory Risk Indicator for Necrotizing Fasciitis), introduced by Wong and colleagues in 2004 [29]. These scoring systems are often implemented during the early stages of care in critically ill patients with H&N NF to monitor disease progression [27,29-32]. In several studies, higher scores have been associated with poorer outcomes and increased mortality [1,33]. However, some reports suggest that the predictive accuracy of certain scores, particularly the LRINEC, may require further refinement [30,32]. For example, in the H&N NF cohort described by Vittetoe and colleagues, poorer outcomes were associated with diabetes mellitus and nfSOFA scores of two or higher, whereas LRINEC scores did not significantly differ between the “poor outcomes” group and the “good outcomes” group [1].

Microbiological cultures, ideally obtained from tissue or fluid samples collected prior to the initiation of antibiotic therapy, frequently reveal polymicrobial aerobic and anaerobic co-infections consistent with an odontogenic or pharyngeal origin; subsequent antimicrobial susceptibility testing further guides targeted therapy [34]. Bedside tissue testing, which is commonly used for the early diagnosis of necrotizing infections in other anatomical regions (such as the trunk, extremities, or external genitalia), is typically not performed in DNI or DNM cases [35]. This omission is primarily due to practical priorities and the inherent procedural limitations of the cervical region.

Imaging and Endoscopic Evaluation

In cases of DNI or DNM, chest radiography may demonstrate pathological features such as tracheal deviation secondary to cervical swelling, mediastinal widening, pneumomediastinum, pneumoperitoneum, or subcutaneous emphysema. Furthermore, it may reveal evidence of pneumonia, pulmonary infiltrates, pleural effusion, and pneumothorax [15,36]. When an odontogenic source is suspected, a panoramic dental radiograph is indicated once the patient’s airway has been adequately secured [9].

Ultrasonography (B-mode US) may be useful in selected DNI cases, particularly for identifying and draining localized liquefied abscesses, or for monitoring the patient’s response to conservative treatment [4]. The US can also detect gas in tissues, although this finding is typically identified by other imaging modalities. Finally, it can demonstrate internal jugular vein thrombus, alongside remote metastatic infectious foci, such as hepatic and splenic abscesses [36].

A computed tomography (CT) scan of the head and neck is the indispensable imaging modality for evaluation of DNIs [9]. The diagnostic gold standard for assessing the complex anatomy of the deep cervical spaces and confirming DNM is the contrast-enhanced multiplanar cervicothoracic CT scan. This examination should be performed provided that there is no imminent airway compromise or after the airway has been secured [6,9]. Diagnostic imaging features of DNM, as described by Scaglione and colleagues [37], include mediastinal, pleural, and/or pericardial fluid collections, with or without gas; increased density of cervical adipose tissue, consistent with cellulitis; muscular infiltration, indicative of myositis; cervical lymphadenopathy; and vascular thrombosis. A cervicothoracic CT scan allows for an accurate assessment of the location and extent of the infection, assists in formulating the therapeutic strategy, enables the evaluation of treatment response, and facilitates the monitoring of disease progression [26]. Postoperative CT surveillance is recommended approximately 48 hours following surgery, or routinely every 48 to 72 hours until clinical improvement is achieved; it should be performed earlier if postoperative deterioration is observed [17,22].

Abdominal CT imaging is indicated if the patient’s clinical condition fails to improve or actively deteriorates despite an unremarkable postoperative cervicothoracic CT scan or if clinical symptoms or signs suggest a possible intra-abdominal infection [17]. However, clinical evaluation of the abdomen may be challenging in many cases, as these patients are often intubated and sedated at this stage of management. Likewise, alternative CT modalities may be required in specific clinical scenarios, such as the utilization of CT angiography for vasculature evaluation.

Magnetic resonance imaging (MRI) may serve as an alternative initial or standalone modality in cases of acute neck infection, as it offers unsurpassed soft-tissue contrast resolution [38]. This high-resolution soft tissue discrimination facilitates detailed visualization of normal tissue architectures (including musculature, glandular structures, and mucosa) while detecting soft tissue and bone marrow edema with high sensitivity and defining the characteristics of fluid collections (e.g., discriminating purulent exudates from non-purulent fluid). Evidence suggests high sensitivity, specificity, and positive predictive value of MRI for neck infections, with the additional benefit of avoiding ionizing radiation exposure [39]. Nevertheless, differentiating necrotizing from non-necrotizing soft-tissue infections on MRI can be challenging due to overlapping radiological features [38,40]. Furthermore, MRI may overestimate the extent of DNI by highlighting non-specific edema and fluid collections, whereas CT provides superior detection of soft-tissue gas [41,42]. Besides, MRI is generally more time-consuming, more susceptible to motion artifacts, and more expensive than a CT scan, rendering it less suitable for emergency diagnostics, particularly in patients presenting with dyspnea, claustrophobia, or restlessness. Finally, certain implanted devices are incompatible with MRI. In situations where vascular complications are suspected, magnetic resonance angiography may provide additional diagnostic value [15].

Endoscopic evaluation, including pharyngoscopy, laryngoscopy, or flexible video-nasopharyngolaryngoscopy, may also be performed when necessary to directly visualize the upper aerodigestive tract [4].

Radiologic evaluation, even when yielding only suspicious findings, is imperative to confirm deep space involvement and refine the clinical picture: it assists in ruling out differential diagnoses and pinpointing the possible primary foci. A key example includes the investigation for cervical spondylodiscitis and osteomyelitis in cases of prevertebral space infection. Furthermore, imaging is essential for identifying potential complications of the disease, including carotid sheath involvement, suppurative jugular thrombophlebitis with associated septic pulmonary emboli, local and remote abscesses, spinal cord compression in prevertebral space infections, pleural effusions, empyema, and pneumothoraces. Table 1 presents a comparison of diagnostic findings and clinical utility between CT and MRI in the evaluation of DNIs and DNM.

**Table 1:** Comparative evaluation of computed tomography and magnetic resonance imaging in the management of deep neck infections and descending necrotizing mediastinitis DNI: deep neck infection; DNM: descending necrotizing mediastinitis

Feature / Modality	Computed Tomography (CT)	Magnetic Resonance Imaging (MRI)
Primary Clinical Status	Indispensable imaging modality for DNI; diagnostic gold standard for DNM	Alternative initial or standalone modality for acute neck infection
Soft-Tissue Contrast	Moderate; provides adequate detail with contrast	Unsurpassed soft-tissue contrast resolution and high-resolution discrimination
Anatomical Visualization	Accurate assessment of the location and extent of infection, bony destruction, and deep space boundaries	Detailed visualization of normal tissue architectures (musculature, glandular structures, and mucosa)
Edema & Fluid Characterization	Clear identification of fluid collections and abscess walls	Detection of soft-tissue and bone marrow edema with high sensitivity; defines fluid collection characteristics (purulent versus non-purulent)
Detection of Soft-Tissue Gas	Superior detection of soft-tissue gas	Less effective in detecting gas
Diagnostic Metrics	High sensitivity for detecting deep neck space abscesses, but lower specificity in differentiating cellulitis from early abscesses	High sensitivity, specificity, and positive predictive value for neck infections
Radiation Exposure	Ionizing radiation exposure	Avoiding ionizing radiation exposure
Diagnostic Accuracy Risks	May miss early-stage infections before a frank abscess forms; can occasionally misidentify dense phlegmon as a drainage-ready fluid collection	May overestimate the extent of DNI by highlighting non-specific edema and fluid collections; shares challenges differentiating necrotizing from non-necrotizing infections due to overlapping radiological features
Vascular Evaluation	CT angiography for rapid assessment of vessel patency, erosion, or thrombosis	Magnetic resonance angiography for when vascular complications are suspected
Practical & Technical Limitations	Highly accessible, fast scan times, and minimally impacted by mild patient movement	More time-consuming, more susceptible to motion artifacts, and more expensive
Emergency & Patient Suitability	Ideally suited for emergency settings and critically ill, unstable patients	Less suitable for an emergency setting, particularly in cases of dyspnea, claustrophobia, or restlessness
Contraindications	Minimal; primarily limited by severe iodine contrast allergy or severe acute renal failure (if contrast is required)	Certain implanted devices (e.g., non-MRI-safe pacemakers, metallic foreign bodies, aneurysm clips) are strictly incompatible

Diagnostic Criteria

Currently, there are no universally established or standardized diagnostic criteria for H&N NF. Consequently, patient diagnosis in the published literature relies on inconsistent criteria, ranging from clinical and radiological findings to histopathological evaluation [25,43]. Conversely, the established diagnostic criteria for DNM, initially defined by Estrera and colleagues [44] in 1983 and further refined by Wheatly and colleagues [18] in 1990, facilitate consistency and validity in clinical assessment. These criteria currently consist of all of the following: clinical evidence of severe cervical/oropharyngeal infection (odontogenic, peritonsillar, or retropharyngeal abscess, Ludwig’s angina, or infection from pharyngeal penetrating trauma); demonstration of characteristic radiographic features of mediastinitis; documentation of necrotizing mediastinal infection during surgery or at postmortem examination or both; and establishment of a clear relationship between the primary oropharyngeal infection and the subsequent development of necrotizing mediastinitis.

Differential Diagnosis

In its early stages, H&N NF may pose diagnostic challenges, as it closely mimics other deep neck space infections. Primary infectious differential diagnoses include uncomplicated cellulitis, peritonsillar or parapharyngeal abscesses, and suppurative lymphadenopathy [25]. Non-infectious diseases can also present with a clinical picture similar to early-stage necrotizing infections; examples include longus colli tendinosis, metastatic lymphadenopathy, Kikuchi lymphadenitis, and neck muscle hematomas. While these alternative clinical entities typically manifest with localized edema, erythema, and pain, they lack the fulminant progression of NF, the soft-tissue crepitus, as well as other gradually developing signs like overt cutaneous changes (bullae, ecchymosis, or frank necrosis) and systemic toxicity. Consequently, maintaining a high index of clinical suspicion, coupled with prompt radiological evaluation, is paramount to accurately differentiate necrotizing disease from these benign mimics.

Regarding mediastinal involvement, the early recognition of progressive respiratory compromise or impending airway obstruction, alongside associated findings such as chest pain, hemodynamic instability, and high fever, can greatly facilitate the prompt diagnosis of DNM. These findings also assist in differentiating DNM from alternative causes of neck swelling, regional lymphadenopathy, and chest symptoms, such as those stemming from acute esophageal pathology or sternotomy infections, thereby guiding the rapid selection of the proper medico-surgical treatment [17,23]. It is highly critical to distinguish suppurative descending mediastinitis from mediastinitis secondary to cardiothoracic surgery via sternotomy, upper esophageal perforation or trauma, ingestion of foreign bodies, or neoplastic malignancy. In these latter conditions, the primary pathology originates within the mediastinum itself and therefore requires different therapeutic approaches and surgical access [26]. Notably, the mortality associated with DNM is reported to be nearly twice as high as that observed in these other forms of mediastinitis [45]. Finally, post-mortem autopsy diagnosis of a necrotizing infection involving the cervical tissues and mediastinum often carries profound medico-legal implications [22].

Diagnostic Challenges and Barriers

Because of the complex regional anatomy of the neck, characterized by the close proximity of the upper airway to other vital structures, the non-specific nature of early infectious symptomatology, and the relative rarity of the condition, the timely diagnosis and appropriate management of H&N NF and DNM remain particularly challenging. Delayed recognition may lead to devastating local and systemic complications, including death [6].

A primary diagnostic obstacle is the limitation of time, as many patients present to tertiary care institutions with a significantly delayed timeline from symptom onset [4,14,23,26]. Consequently, individuals often present with advanced disease, manifest sepsis, or multiple organ dysfunction, necessitating immediate clinical evaluation and urgent therapeutic intervention.

A secondary challenge stems from the inconspicuous initial presentation of NF [3,4,8,19,25,33]. Clinicians must identify the potential for such infections through a high index of clinical suspicion, even when presenting signs remain vague. This is especially true for patients with a history of recent head or neck infections, as incipient NF can easily be misdiagnosed as uncomplicated cellulitis or a localized DNI. Physical examination demonstrates limited sensitivity, and DNIs are rarely evident upon palpation during early phases. Owing to these subtle initial symptoms, the diagnosis of DNM is frequently delayed, with identification often occurring only after the onset of sepsis. In any case, DNM should be strongly suspected in patients presenting with oropharyngeal inflammatory findings and fever whose general condition deteriorates rapidly alongside severe respiratory distress and septic shock [10].

Risk assessment focuses primarily on patients with known predisposing comorbidities. Established risk factors include diabetes mellitus; underlying cardiovascular, chronic pulmonary, renal, and neurological disorders; as well as malignant neoplasms and hepatic cirrhosis. Furthermore, conditions that inherently compromise tissue oxygenation, such as morbid obesity, peripheral artery occlusive disease, or a history of regional neck radiotherapy, serve as critical compounding factors [1,3,4,7]. Primary or secondary immunodeficiencies, such as chronic alcoholism or immunoglobulin deficits, also place patients at a higher risk. Diabetes mellitus represents the most frequent comorbidity for these infections. Nevertheless, a noteworthy cohort of patients presents with no identifiable predisposing comorbidities. Epidemiologically, NF most commonly afflicts middle-aged adults, demonstrating no clear predilection with respect to gender, race, or geographic distribution [9].

Specific patient groups demand heightened clinical vigilance. Oncologic patients with H&N NF frequently exhibit highly atypical presentations, wherein early necrotizing symptoms may be easily misattributed to tumor progression or treatment-related toxicities [25]. Similarly, immunocompromised patients may manifest with markedly attenuated clinical signs and deceptive laboratory parameters; in these vulnerable populations, diagnostic delays precipitously accelerate the trajectory toward patient mortality. These key observations underscore the need for a low threshold for diagnostic imaging in these subgroups.

It is critical to acknowledge that laboratory parameters, scoring algorithms, and radiological modalities provide only suggestive evidence; they lack absolute diagnostic sensitivity and may fail to correlate linearly with real-time disease progression [1,4,5]. Optimizing patient outcomes necessitates a holistic multimodal assessment, integrating objective clinical evaluation with laboratory and imaging results. Serial re-evaluations of all these parameters can improve prognosis. Even if primary surgical management has been executed, suboptimal cervical incisions can lead to insufficient surgical drainage, resulting in persistent post-surgical sepsis that may remain unrecognized until reaching an advanced stage. Secondary complications must also be actively evaluated during the diagnostic process because they directly affect the overall clinical outcome. Consequently, diagnostic evaluation must be treated as a dynamic and continuous process, requiring repeated clinical and imaging reassessments to promptly identify and control residual, persistent, or newly developing infectious foci.

Finally, resource-limited settings present a profound diagnostic challenge. In the absence of advanced radiological infrastructure, the surgeon must decisively weigh the available clinical indicators and assess the necessity of diagnostic exploratory surgery.

To address these diagnostic bottlenecks, advanced computational methods are increasingly being investigated as potential adjunct tools. Driven by recent progress in artificial intelligence (AI), machine learning algorithms such as k-nearest neighbors (k-NN) and eXtreme Gradient Boosting (XGBoost), alongside dimensionality reduction techniques like t-distributed stochastic neighbor embedding (t-SNE) and Principal Component Analysis (PCA), have been explored for medical classification and prognostic modeling [46]. By constructing predictive pipelines that integrate multimodal clinical, laboratory, and radiological parameters, these machine learning approaches may help model complex, high-dimensional datasets that traditional scoring systems frequently fail to capture. Extensive and well-structured data on critically ill patients provides an ideal foundation for developing and validating these machine learning-based risk prediction models [47]. Proponents suggest that such models could assist clinicians in identifying patients with DNIs at high risk of rapid progression to DNM. However, their current clinical utility remains bound by the quality of input data, the need for prospective validation, and the crucial, serial, hands-on clinical re-evaluation. If validated, such algorithms could successfully support prompt decision-making and help guide timely, life-saving procedures such as early tracheostomy when indicated [46]. Table 2 summarizes diagnostic Indicators, clinical manifestations, and evaluation modalities in DNM.

**Table 2:** Summary of diagnostic clues in descending necrotizing mediastinitis AI: artificial intelligence; APACHE II: Acute Physiology and Chronic Health Evaluation II; ARDS: acute respiratory distress syndrome; CT: computed tomography; DNI: deep neck infection; DNM: descending necrotizing mediastinitis; LRINEC: Laboratory Risk Indicator for Necrotizing Fasciitis; MRI: magnetic resonance imaging; SOFA: Sequential Organ Failure Assessment; US: ultrasound

Diagnostic Category	Specific Clues & Clinical Indicators
Initial Signs & Symptoms	Pain, neck swelling, fever, oropharyngeal edema, erythema, subcutaneous emphysema, pneumomediastinum, respiratory distress, ARDS, hemodynamic instability, multiple organ dysfunction and failure, septic shock
Clinical Progression	Rapid clinical deterioration
Patient History	May be preceded by recent dental procedures, surgeries, or interventions
Predisposing Factors	Conditions associated with reduced tissue oxygenation or immune dysfunction
Differential Diagnosis	Exclusion of other underlying causes of neck swelling or mediastinitis
Imaging Findings	Chest X-rays; ultrasound (US): small abscess drainage or surveillance; CT scan: modality of choice for diagnosis and surveillance; MRI: for acute DNIs and assessment of vascular involvement
Endoscopic Evaluation	Pharyngoscopy and laryngoscopy to evaluate and protect airways
Microbiology	Culture results from samples collected immediately at admission
Lab Findings & Scoring	Routine parameters and laboratory-based scoring systems (APACHE II, SOFA, LRINEC)
Advanced Tools	Predictive models and algorithms utilizing AI
Post-Intervention Plan	Postoperative repeated clinical and imaging reassessment for survivors

Management and outcomes

There is general agreement that the management of DNI and DNM should be tailored to the individual patient and provided promptly, aggressively, and within a collaborative framework. This multidisciplinary team typically encompasses specialists in infectious diseases, intensivists, thoracic surgeons, otorhinolaryngologists, oral and maxillofacial surgeons, general surgeons, anesthesiologists, radiologists, and immunologists [3,14]. Emergently securing the airway to maintain patency and ensure adequate respiratory function is the highest priority in critically ill patients and is best achieved within an intensive care unit setting [8,48,49]. Therapeutic strategies, frequently structured through various treatment and monitoring protocols and algorithms, rely on the continuous and nearly simultaneous support of vital functions, appropriately adjusted antimicrobial therapy, and repeated surgical debridement and drainage [1,15,49]. Specific treatment details, however, fall outside the scope of this review and are not discussed herein.

Despite widely accepted treatment recommendations, postoperative morbidity and mortality in DNM remain substantial. This persistent mortality is mainly attributed to acute airway compromise or severe sepsis and its complications, including ARDS, cardiac tamponade, acute renal failure, pneumonia, chylothorax, and stroke [17]. Mortality remains exceptionally high among patients who are admitted with established sepsis, and septic shock at admission has been consistently identified across several studies as an independent predictor of mortality [23]. In line with most other studies, two recent large clinical series highlighted the detrimental effects of delayed intervention on patient survival with DNM [11,27]. However, the historically extremely high mortality rates of DNM, which stood at up to 70% during the mid-to-late 20th century [17,23,34], have been substantially mitigated. Current studies report a reduced mortality range of 11% to 40%. This decline likely reflects the cumulative beneficial impact of the advancements in anesthesia and clinical care, the introduction of modern intravenous antibiotics, the widespread availability of CT imaging, and the more aggressive surgical interventions combined with multidisciplinary management paradigms [1,23,34]. Table 3 outlines mortality rates associated with DNM reported over the last 15 years.

**Table 3:** In-hospital mortality of H&N NF/DNM during the last 15 years Selected studies on H&N NF and CNF are also included. Mortality data across all included studies were derived from retrospective analyses of case series. Studies are listed in strict chronological order based on publication year. *Overall mortality regardless of timeframe; **Mean age unless otherwise stated, and relevant to the disease comorbidities. CNF: craniofacial/cervical necrotizing fasciitis; DNI: deep neck infection; DNM: descending necrotizing mediastinitis; H&N NF: head and neck necrotizing fasciitis

Author, Year (Ref)	Study Period	Country / Institution	Patients (n)	Pathology	Mortality (%)*	Criteria / Classification	Mean Age (n) & Comorbidities (%)**
Kocher 2012 [26]	1999– 2011	Switzerland (University Hospital Bern)	17	DNM	5.9	Estrera classification	53.0% comorbidities
Dajer-Fadel 2014 [50]	2005– 2012	Mexico (General Hospital of Mexico)	60	DNM	35.0	Clinical, laboratory, and imaging findings, and a clear relation between DNI and mediastinitis	41.2 ± 14.7 years 46.7% comorbidities
Cruz Toro 2014 [51]	2005– 2010	Spain (Hospital de Bellvitge)	6	CNF	0.0	Clinical, imaging & surgical findings	58 years 0.0% comorbid
Ma 2019 [23]	2010– 2017	China (Peking Union Medical College)	16	DNM	18.75	Clinical findings, cervicothoracic CT, and a clear relation between DNI and mediastinitis	54.9 ± 14.3 years 62.5% comorbidities
Wu 2021 [52]	2006– 2019	China (Wenzhou Medical University)	9	DNM	0.0	Estrera classification	44.33 ± 13.54 years
Escobedo 2021 [53]	1987– 2018	Spain (Oral & Maxillofacial Service)	7	DNM	14.2	Estrera classification	30.4 years
Gehrke 2022 [54]	2009– 2019	Germany (University of Würzburg)	45	DNM	8.89	Clinical and imaging findings	61.33 years 60% comorbidities
De Palma 2022 [11]	2002– 2020	Italy (University of Bari)	15	DNM	0.0	Clinical signs and imaging	49.07 ± 14.92 years
Reuter 2023 [10]	1997– 2018	Germany (University of Freiburg)	88	DNM	9.1	Estrera classification	54.7 years 51.0% comorbidities
Vittetoe 2023 [1]	2006– 2021	USA (Vanderbilt University)	33	H&N NF	6.06	Pathology and operative findings	66.6% comorbidities
Venegas Pizarro 2024 [8]	2019– 2022	Spain (Hospital de la Santa Creu)	7	DNM	0.0	Estrera classification	50 years 42.8% comorbidities
Zhao 2023 [55]	2012– 2022	China (Peking Union Medical College)	31	DNM	25.8	Estrera classification	Median age: 57 years
Uchikov 2024 [14]	2018– 2021	Bulgaria (Med University of Plovdiv)	4	DNM	75.0	Estrera classification	Age range: 23–33 years. No comorbidities
Ho 2024 [34]	2016– 2021	Taiwan (Chang Gung Memorial Hosp)	21	DNM	9.5	Clinical and radiographic findings and a clear relation between DNI and mediastinitis	56.7 ± 22.2 years
Ramos-Hinojosa 2025 [27]	2017– 2024	Mexico (General Hospital of Mexico)	45	DNM	33.3	Clinical and imaging criteria	49.8 ± 14 years
Pupić-Bakrač 2025 [56]	2006– 2023	Croatia (Multicenter)	22	H&N NF	9.1	Clinical, imaging & surgical findings	Data not specified
Parjiea 2025 [57]	2008– 2022	Germany (University of Erlangen)	22	DNM	9.0	Estrera classification	60 ± 9 years
Deng 2025 [58]	2014– 2023	China (Zunyi Medical University)	30	DNM	36.6	Estrera classification	Median age: 53 years
Chiyo 2026 [59]	2004– 2024	Japan (Chiba University Hospital)	37	DNM	8.1	Clinical and imaging findings	64.1 years

Study limitations

Regarding limitations, this narrative review is restricted foremost by its reliance on published terminology for the identification of cases within the literature. Consequently, only studies explicitly identifying cases as NF and descending mediastinitis within the searched databases were captured. Furthermore, only cases with operative and pathology findings consistent with these infections were included. This methodology makes it highly probable that many relevant cases of H&N NF and DNM were missed in our search strategy due to imprecise or incomplete reporting in the primary literature. Additionally, the retrospective nature of the included publications introduces significant heterogeneity regarding data quality and the degree of detail reported. The absence of randomized controlled trials in this field and a potential publication bias favoring positive outcomes must also be acknowledged. Moreover, this review evaluated literature published over a 15-year period (2011-2026). Trends in the diagnosis and treatment for H&N NF and DNM have inevitably evolved over this timeframe; these changes pose a lack of homogeneity regarding the clinical management paradigms utilized by the various attending physicians. Further, because these conditions represent rare disease processes, the sample sizes of most included articles were insufficient to conduct multivariate analyses, thus limiting the ability of the primary authors to draw conclusive statistical evidence. Finally, since our search strategy was limited to specific databases and English-language literature, relevant articles published in less well-known databases were inevitably omitted.

These limitations highlight crucial areas for future investigation, particularly in a prospective setting where standardized clinical data can be gathered to fully capture the natural history of the disease and establish optimal diagnostic algorithms. Future prospective, large-scale, multicenter studies with extensive patient cohorts are required to provide deeper clinical insights into these rare conditions. Such high-quality data will be essential to formally validate diagnostic scores, imaging and pathology criteria, and the potential integration of artificial intelligence in clinical decision-making.

## Conclusions

Because highly virulent pathogens can spread through or along fascial planes, any orofacial and cervical infection can potentially involve the entire mediastinum. Despite better knowledge of the natural history of the disease, improvements in diagnostic imaging, particularly with CT scans, and more effective medico-surgical interventions, DNM remains fatal in at least one-quarter of patients in most studies. Clinical vigilance and sound judgment are critical to halting the fulminant and potentially deadly progression of the disease. The cornerstones of effective DNM management are early recognition of the disease, a coordinated multidisciplinary approach, and the prompt implementation of aggressive modern medical and surgical therapies.
